# A Dictionary of Chemistry

**Published:** 1821-09-01

**Authors:** 


*A Dictionary of Chemistry, on the Basis of Mr. Nicholson's; in which the Principles of the Science are investigated anew, and its Applications to the Phenomena of Nature, Medicine, Mineralogy, Agriculture, and Manufactures, detailed; with an Introductory Dissertation, containing Instructions for converting the alphabetical Arrangement into a systematic Order of Study.* By Andrew Ure, M.D. Professor of Chemistry and Natural Philosophy in the Andersonian Institution of Glasgow. Octavo, 14 plates, pp. 1060. London, 1821.

Lexicographical systems of science and philosophy, from the peculiarities of their nature and construction, are obviously insusceptible of analytical concentration. Nevertheless, we shall endeavour to arrange a brief exposition of the more important of Dr. Ure's original essays; and, of these, shall select such only as possess relation to the objects of chemical and medical researches.

I. Chemical Sketches. *Chemistry,* in the language of Dr. Ure, is "the science which investigates the composition of material substances, and the permanent changes of constitution which their mutual actions produce." This general definition being premised, we proceed to direct the attention of our readers to some of the articles which relate to chemical science.

*Acids.* Dr. Ure embraces the chloridic doctrine of acidification, and regards it as being confirmed by the best experimental and inductive evidence. Finding the classification of other writers to be in many respects exceptionable, he proposes a new systematic distribution of the acids, wherein his chief object is to groupe together such of these substances as have analogous properties or composition. He places them in two general divisions. 1st. Acids from inorganic nature, or which are procurable without having recourse to animal or vegetable products. 2d. Acids elaborated by means of organization. The first groupe is subdivided into three families—oxygen acids—hydrogen acids—and acids destitute of both these supposed acidifiers. The acids of the last division are all decomposable at a red heat, and afford generally carbon, hydrogen, oxygen, &c. and in some few cases also nitrogen. The general properties of the acids are thus stated.

"1st. The taste of these bodies is for the most part sour, as their name denotes; and in the stronger species it is acrid and corrosive. 2d. They generally combine with water in every proportion, with a condensation of volume and evolution of heat. 3d. With a few exceptions, they are volatilized or decomposed at a moderate heat. 4th. They usually change the purple colours of vegetables to a bright red. 5th. They unite in definite proportions, with the alkalis, earths, and metallic oxids, and form this important class of salts. This may be reckoned their characteristic and indispensable property."

Much new and valuable information will be found in the articles where different acids are described. Among others, those on the arsenious, carbonic, fluoric, hydriodic, chloriodic, muriatic, nitric, prussic, or hydrocyanic, acids. Chlorocyanic, pyrolignous, sulphuric, and the hyposulphurous acids, contain details and tables by Dr. Ure, which are in a high degree interesting and instructive.

*Alkalis.* These are arranged, in the present work, into three classes. "1. Those which consist of a metallic basis combined with oxygen. These are three in number, potash, soda, and lithia. 2. That which contains no ammonia. 3. Those containing oxygen, hydrogen, and carbon: in this class are the vegetable alkalis. Besides neutralizing acidity, and thereby giving birth to salts, the first four have the following properties. 1. They change the purple colour of many vegetables to a green, the red to a purple, and the yellow to a brown. If the purple have been reddened by an acid, the alkalis restore the purple. 2. They possess this power on vegetable colours *after* being saturated with carbonic acid, by which criterion they are distinguishable from the alkaline earths. 3. They have an acrid and urinous taste. 4. They are powerful solvents or corrosives of animal matter, with which, as well as with oils in general, they combine so as to produce neutrality. 5. They are decomposed or volatilized at a strong red heat. 6. They combine with water in every proportion, and also largely with alcohol. 7. They continue to be soluble in water when neutralized with carbonic acid; while the alkaline earths thus become insoluble."

*Attraction.* This forms the subject of a disquisitive sketch containing many new and remarkable observations. We were much pleased to find here, for the first time, Dr. Young's tables of elective attractions introduced into an English system of chemistry, and accompanied with his admirable remarks on the sequences of double decompositions.

*Calcium*, the metallic basis of lime, has been investigated by Dr. Ure with his characteristic zeal. From his promised experiments, synthetical and analytical, we anticipate a further illustration of this difficult subject.

*Caloric*. An elaborate memoir from Dr. Ure's own pen, has been devoted to the illustration of this most important branch of physical science. Having shewn "how little room there is to pronounce dogmatic decisions on the abstract nature of heat," he goes on to say—

"If the essence of the cause (caloric) be still involved in mystery, many of its properties and effects have been ascertained, and skilfully applied to the cultivation of science, and the uses of life. We shall consider them in the following order:—1. Of the measure of temperature. 2. Of the distribution of heat. 3. Of the general habitudes of heat with the different forms of matter."

An astonishing patience of research, an intimate acquaintance with all the thermological doctrines, an appropriate use of inductive ratiocination, and an elegant perspicuity of diction by no means common in scientific literature, are manifest throughout the discussion of these general heads. The monograph is concluded in these terms:—

"I have thus completed," says the author, "what I conceive to belong directly to caloric in a chemical dictionary. Under *alcohol attraction*, *blow-pipe*, *climate*, *combustion*, *congelation*, *digester*, distillation, electricity, *gas*, *light*, *pyrometer*, *thermometer*, *water*, some interesting correlative facts will be found."

*Chlorine*, with its dependants, chloro-carbonous and chlorous acids, furnish the author with an opportunity of panegyrizing Sir Humphrey Davy, and of ascribing a complete ascendancy to the new doctrines of that distinguished philosopher. These papers contain a perspicuous summary of the chloridic system: they are written with great spirit, and bear ample testimony to the degree of Dr. Ure's professional zeal and the indefatigability of his research. We feel prompted to transcribe his prefatory observations.

"The introduction of this term," (chlorine,) he begins, "marks an era in chemical science. It originated from the masterly researches of Sir H. Davy on the oxymuriatic gas of the French school, a substance which, after resisting the most powerful means of decomposition which his sagacity could invent or his ingenuity apply, he declared to be, according to the true logic of chemistry, an elementary body, and not a compound of muriatic acid and oxygen, as was previously imagined, and as its name seemed to denote. He accordingly assigned to it the term chlorine, descriptive of its colour; a name now generally used. The chloridic theory of combustion, though more limited in its applications to the chemical phenomena of nature than the antiphlogistic of Lavoisier, may justly be regarded as of equal importance to the advancement of science itself. When we now survey the Transactions of the Royal Society for 1808, 1809, 1810, and 1811, we feel overwhelmed with astonishment at the unparalelled skill, labour, and sagacity, by which the great English chemist, in so short a space, prodigiously multiplied the objects and resources of the science, while he promulgated a new code of laws, flowing from views of elementary action, equally profound, original, and sublime. The importance of the revolution produced by his researches on chlorine, will justify us in presenting a detailed account of the steps by which it has been effected."

*Combustion* is the subject of a memoir, than which we have not seen a better in any chemical system. It is distributed into six general heads, under which the phenomena of combustion are considered. "1. The temperature necessary to inflame different bodies. 2. The nature of flame, and the relation between the light and heat which compose it. 3. The heat disengaged by different combustibles in burning. 4. The causes which modify and extinguish combustion, and of the safe-lamp. 5. Invisible combustion. 6. Practical inferences."

In the course of this article Sir H. Davy's discovery of the "safety lamp" and the "noble truths first revealed by him concerning the mysterious process of combustion," are made the theme of eloquent and triumphant eulogy. The "practical inferences" conclude with this aphorismal remark: "*Finally, we may establish it as an axiom, that combustion is not the great phenomenon of chemical nature; but an adventitious accidental accessory to chemical combination, or decomposition; that is, to the internal motions of the particles of bodies, tending to arrange them, in a new chemical constitution.*"

*Congelation and Dew.* Many ingenious observations and experiments are enumerated in these articles. With respect to the formation of dew, our author, following Dr. Wells, regards this proposition as established by a "copious induction of facts:"—"*That bodies become colder than the neighbouring air* BEFORE *they are dewed.*" The cold, therefore, which Dr. Wilson and Mr. Six conjectured to be the *effect* of dew, now appears to be its *cause*. But what makes the terrestrial surface colder than the atmosphere? The radiation or projection of heat into free space. Now, different bodies project heat with very different degrees of force. In the operation of this principle, therefore, conjoined with the powers of a concave mirror of cloud, or any other awning, to reflect or throw down again those calorific emanations which would be dissipated in a clear sky, we shall find a solution of the most mysterious phenomena of dew."

*Electricity*, in its modern augmentation, seems to comprehend almost every change of the corpuscular world, however minute and mysterious, as well as the long recognized and magnificent motions of the atmosphere. Dr. Ure considers the electrical phenomena under a fourfold arrangement. "1. Of the EXCITEMENT of electricity, or the various means by which the electrical equilibrium is distributed. 2. Of the *two electricities*. 3. Of the distribution of electricity. 4. Of the voltaic battery and its EFFECTS; calorific or igniting; and decomposing, or the chemical agencies of electricity." "Concerning the nature of the electrical essence," he adds, "we are equally in the dark as concerning the nature of caloric. The phenomena may be referred in both cases, either to a peculiar fluid whose particles are endowed with innate idio-repulsive powers, or to a peculiar affection of the molecules of common matter."

The memoirs on electricity and galvanism belong to the highest style of scientific description. They are, in all respects, ingenious, curious, and useful. The article on *chemical equivalents* consists of accurate, experimental, and philosophical observations.

*Eudiometer*. Under this head, Dr. Ure introduces the description of an apparatus for the analysis of gazeous matter by explosion. It was invented by himself about three years ago, and is a simple instrument, "in which the atmospheric air, the most elastic and economical of all springs, is employed to receive and deaden the recoil."

"It consists," says he, "of a glass syphon, having an interior diameter of from 2-10ths to 4-10ths of an inch. Its legs are of nearly equal lengths, each being from six to nine inches long. The open extremity is slightly funnel-shaped; the other is hermetically sealed; and has inserted near it, by the blow-pipe, two platina wires. The outer end of the one wire is incurvated across, so as nearly to touch the edge of the aperture: that of the other is formed into a little hook, to allow a small spherical button to be attached to it, when the electrical spark is to be transmitted. The two legs of the syphon are from one-fourth to one-half inch asunder. The sealed leg is graduated, by introducing successively equal weights of mercury from a measure-glass tube. Seven ounces troy and 66 grains occupy the space of a cubic inch; and $34\frac{1}{4}$ grains represent $\frac{1}{100}$ part of that volume. The other leg may be graduated also, though this is not necessary. The instrument is then finished."

The manner of using it is simple and described with much perspicuity; but we cannot afford space for its insertion.

*Gas.* Dr. Ure arranges his general observations on the gases into four heads. In the first section is a "general table of gaseous bodies," and under the fourth, a "table of reduction of gaseous volumes, for variations of temperature above or below 60°," both by Dr. Ure's own pen. His division is this:—"1. Tabular views of the densities and combining ratios of the gases. 2. A description of their general habitudes with solids and fluids. 3. An account of the principal modes of analysing gaseous mixtures. 4. Of gasometry, or the measurement of the density and volume of gases."

*Iodine.* From a series of facts experimentally determined, Dr. Ure finds himself warranted in concluding iodine to be *an undecomposed body.*

"In its specific gravity, lustre, and magnitude of its prime equivalent," he assures us, "it resembles the metals; but in all its chemical agencies it is analogous to oxygen and chlorine. It is a non-conductor of electricity, and possesses, like these two bodies, the negative electrical energy with regard to metals, inflammable, and alkaline substances; and hence, when combined with these substances in aqueous solutions, and electrized in the voltaic circuit, it separates at the positive surface. But it has a positive energy with respect to chlorine; for, when united to chlorine, in the chloriodic acid, it separates at the negative surface. This likewise corresponds with their relative attractive energy, since chlorine expels iodine from all its combinations. Iodine dissolves in carburet of sulphur, giving, in very minute quantities, a fine amethystine tint to the liquid."

We shall revert to the medical qualities of iodine.

*Light,* "the agent of vision," constitutes the subject of a very philosophical sketch. "The physical affections of light," says the author, "are foreign to this work. Its chemical relations may be conveniently referred to four heads."

"1. Of the mean refractive and dispensive powers of different bodies. 2. Of the action of the different prismatic colours on chemical matter. 3. Of the polarisation of light. 4. Of the absorption and disengagement of light or phosphorescence." Under the last head, Dr. Brewster's tabular view of mineral phosphorescence is introduced.

*Prussine.* Dr. Ure objects to its synonima *cyanogen,* the producer of blue. "The same reason," says he, "which tends to the term cyanogen, would warrant us in calling it leucogen, erythrogen, or chlorogen, for it produces white, red, or green, with other metals, if it produce blue with iron." He prefers the epithet *prussine*, and describes its chemical qualities in a neat analytical sketch. But we must not lose sight of the second part of our arrangement.

II. Medical Chemistry. Connected with this department of science, the Dictionary contains many articles in which are inserted the results of much experimental research. Of these we shall notice a few, chiefly in an abstractive form.

*Arsenious Acid.* Under this head will be found whatever is known of the nature, action, effects, treatment, and tests of arsenical disease. The medical student will find in it a concise account of the processes by which that substance may be obtained for therapeutical purposes, together with rules for administering its different preparations, and a description of its influences on the living system.

*Adipocere.* In the description of this substance, and under the articles *Bezoar, Gall,* and *Intestinal Concretions,* is enumerated all the knowledge of these singular compositions which modern chemistry affords. Among other ingenious speculations, the following are introduced under the first of these heads.

"In the human colon, solid masses of fat are sometimes met with in a diseased state of that canal, and are called *scybala*. A description and analysis by Dr. Ure of a mass of ambergris, extracted in Perthshire from the rectum of a living woman, were published in a London Medical Journal,* in September 1817. There is a case communicated by Dr. Babington, of fat formed in the intestines of a girl, four and a half years old, and passing off by stool. Mr. Brande found, on the suggestion of Sir. E. Home, that muscle digested in bile, is convertible into fat, at the temperature of about 100°. If the substance, however, pass rapidly into putrefaction, no fat is formed. Fæces voided by a gouty gentlemen after six days' constipation, yielded, on infusion in water, a fatty film. This process of forming fat in the lower intestines by means of bile, throws considerable light upon the nourishment derived from clysters, a fact well ascertained, but which could not be explained. It also ac-

* See, in the monthly series of the Medico-Chirurgical Journal, Vol. iv. p. 177, "An Account of a Morbid Concretion discharged from the Rectum of the Human Female, and in its Chemical Characters closely resembling Ambergris, with historical Remarks, by James Kennedy, M.D of Dunning, Perthshire, July 1817.

counts for the wasting of the body which so invariably attends all complaints of the lower bowels. It accounts, too, for all the varieties in the turns of the colon, which we meet with in so great degree in different animals. This property of the bile explains likewise the formation of fatty concretions in the gall-bladder so commonly met with, and which, from these experiments, appear to be produced by the action of the bile on the mucus secreted in the gall-bladder; and it enables us to understand how want of the gall-bladder in children, from mal-formation, is attended with excessive leanness, notwithstanding a great appetite, and leads to an early death. Fat thus appears to be formed in the intestines, and from thence received into the circulation, and deposited in almost every part of the body. And, as there appears to be no direct channels by which any superabundance of it can be thrown out of the body, whenever its supply exceeds the consumption, its accumulation becomes a disease, and often a very distressing one."

*Calculus.* Dr. Marcet's arrangement of urinary concretions is here adopted. Their constituent and distinctive principles, the morbid effects they produce, and the best means of counteracting their influences on the health of man, are comprehensively enumerated.

*Galvanism.* The physiologist will find in this article a detail of the galvanic phenomena exhibited in the University of Glasgow, the 4th of November, 1818, on the body of Clydesdale, the murderer. But as these experiments, which are of no common kind, have been published elsewhere, we shall pass them over.

*Iodine* has of late been recommended for the cure of bronchocélé, by Dr. Coindet of Geneva, who administered it in doses of three grains a day, with complete success. Dr. Kennedy, of Dunning, has tried it in a case of that disease, and gradually pushed it from 2-4-8-10-12-15 to 18 grains in the day, but without the least advantage. The medicine had been prepared by Dr. Ure, and was of the best quality. Two ounces of it were used in the space of eighty days, during which time the tumour had considerably increased. On two occasions vomiting was induced, but no other inconvenience was experienced, nor was any other remedy employed at any period of the treatment. Into this article of the Dictionany, Dr. Ure has introduced the experiments*

---

* Those of Dr. Coindet will be found in the "Annals de Chimie," for September 1820; in the "Journal de Pharmacie," for October; and in the "London Medical Repository," for December, of the same year.

of M. Orfila, for the purpose of ascertaining the effects of iodine on the assimilative organs of animals.

We shall here introduce the following abridged account of Dr. Coindet's farther experience of iodine, from the Bibliotheque Universelle, for April 1821, as drawn up by the editor of the Medical Intelligencer, in the 20th number of that Journal.

"The introduction of iodine into the materia medica, for the specific purpose of curing bronchocele, is due to Dr. Coindet, a very experienced practitioner of Geneva. In a former memoir, published by this gentleman, he expressed a wish, that, by the joint efforts of physicians and chemists, we should one day succeed in procuring a more suitable preparation of this substance than the one now in use,—to which many objections had been urged by some practitioners.

"In the present memoir he expresses his conviction that the hydriodate of potash, used externally, will answer the object required; and in support of his doctrine, he relates some cases in which this preparation of iodine was ussd topically, with complete success, for the removal of bronchocele and scrofulous swellings.

"Dr. C. directs a pomatum to be prepared for the above purpose, consisting of half a drachm of hydriodate of potash and one ounce and a half of purified hog's lard. Frictions are then made with a quantity of this preparation, of the size of a nutmeg, morning and evening, on the goître and indurated glands, whether scrofulous or situated on the breast. Occasionally, the frictions are to be practised in the course of the lymphatics, and continued till the pomatum is completely absorbed.

"'Une dame agée de 28 ans portoit depuis long-temps un goître volumineux dans le lobe droit, mais bien plus encore dans le lobe gauche du corps thyroide. Il s'étoit considerablement accru il y a trois ans pendant une grossesse. Je jugeai que ce n'étoit qu'une augmentation de volume sans lésion organique. Ce goître altéroit la voix et gênoit la respiration. Après huit jours de frictions les tumeurs étoient sensiblement plus molles, la peau étoit devenue plus épaisse et plus lâche; après quinze jours la diminution étoit encore plus considérable; le goître étoit divise en plusieurs petits lobules très-distincts les uns des autres; au bout d'un mois il a entièrement disparu, la voix et la respiration sont redevenues naturelles, sans que la malade ait éprouvé aucun autre effet sensible de l'action de ce remède.'

"Twenty-two other patients, afflicted with the same malady, were treated much in a similar manner; one half of whom have been completely cured, and the remainder considerably relieved. Dr. Coindet observed, on these occasions, that the iodine, thus thrown into the system by absorption, produced exactly the same beneficial results, as when taken internally; and when no organic lesion is present, the disease of the lymphatic system seems to be acted upon by the iodine, applied externally, with an energy equal to that attributed to the internal remedy.

"In none of the cases in which this application was used, did there appear any untoward effect, such as the iodine, taken internally, is known to have given rise to; though Dr. Coindet thought it necessary to use as much precaution as if he had administered the medicine internally. The author takes this opportunity of remarking, that many local auxiliaries should be resorted to in the case of goître, by which its removal or cure will be greatly accelerated: amongst these, he reckons leeches and emollient fomentations.

"Dr. C. next tried the hydriodate of potash, as a topical application in scrofulous indurated glands; and the success he obtained was beyond his expectation. He, however, prefers, in such cases, the solution of what he calls the induretted hydriodate of potash, taken internally. The following indications of two successful cases will be read with interest:—

"'Une jeune fille agée de dix-sept ans portoit depuis quinze mois sous l'angle de la mâchoire et le long du cou des paquets de glandes scrophuleuses, dont une d'elles, la plus basse restoit ulcérée. On avoit inutilement fait un grand nombre de remèdes; je prescrivis une solution d'hydriodate de potasse iodure, dans l'espace de six semaines toutes les glandes se sont dissipées suivant la marche que je viens d'indiquer, excepté celle qui étoit ulcérée. Une fistule pénétrant dans son centre a nécessité un traitement chirurgical pour compléter la guérison. Une autre jeune fille agée de quatorze ans portoit depuis six mois le long du cou un paquet de glandes engorgées; on avoit inutilement fait tous les remèdes géneraux et locaux indiqués en pareil cas; dans l'espace d'un mois l'usage de la solution d'hydriodate de potasse ioduré a suffi pour la guérir.'

"In some few instances the medicine, administered both internally and externally, seemed to fail.

"It does certainly appear that iodine is a most powerful agent, and one which possesses a specific and stimulating power over the lymphatic system. As such, it might perhaps be given alternately, or in combination, with mercury. In enlargements of the ovaria, one would think it a useful remedy; but care must be had not to administer it where fever is present, or during the period of excitement.

"For the information of those who may feel disposed to give this remedy a trial, we have deemed it proper to subjoin a formula for the preparation of the hydriodate of potash, with which we have been favoured by Dr. Granville, who begs us to add, that this salt is found ready formed in the kelp for the preparation of soda.

"Make a solution of caustic potash, add a sufficient quantity of iodine, and shake the bottle well: the water is thus decomposed—iodic and hydriodic acid are formed, each of which combines with a proportion of the potash,—the former giving rise to an iodate which is little soluble, and consequently is precipitated,—while the latter forms the hydriodate of which we are in search, and which is highly soluble. The liquid containing it, is then to be filtered, and the residue washed with alcohol, of the density of 0.82, so as to obtain another portion of the hydriodate—to be added to the former liquid, which may be set to crystallize. The salt is deliquescent, and has a slight yellowish tinge: it consists of 100 of hydriodic acid, and 37.426 of potash.'

"Thenard observes, that by the process of crystallization, as well as by desiccation, the hydriodate of potash is changed into an ioduret of potassium. If so, the salt employed by Coindet must need become a hydriodate during its trituration with the hog's lard,—the hydrogen of which it attracts, to form hydriodic acid." *Med. Intel. p.* 368.

*Poisons.* This is a brief toxicological sketch. Its concluding paragraph contains this information. "Dr. Lyman Spalding, of New York, announces in a small pamphlet, that for above these fifty years the *scutellaria laterifolia* has proved to be an infallible means for the prevention and cure of the hydrophobia, after the bite of rabid animals. It is better applied as a dry powder than fresh. According to the testimonies of several American physicians this plant afforded perfect relief in above a thousand cases, as well in the human species, as in the brute creation, dogs, swine, and oxen." This assertion is, in fact, totally unfounded.

*Magnesia.* In the article on this primitive earth are related the remarkable case published by Mr. Brande in the Journal of Science and the Arts, and another "in which not only large quantities of a concretion of a similar description were voided, but upon examination after death, which took place perhaps six months after any magnesia had been taken, a collection, supposed to be from four to six pounds, was found imbedded in the head of the colon, which was of course much distended."

*Respiration.* Relating to this function of animals, we find the following hint:—"It is probable that the quantity of carbonic acid, produced in the lungs, varies in different animals, and in the same individual in different circumstances. The change of the blood, from the purple venous to the bright red arterial, seems owing to the discharge of the carbon. An ordinary sized man consumes about 46 thousand cubic inches of oxygen *per diem;* equivalent to 125 cubic feet of air. He makes about twenty respirations in a minute; or breathes twice for every seven pulsations" of his arterial vessels.

*Vegetable Alkalis.* The effects of some of these on the living system may now be noticed. 1. *Morphia,* the narcotic principle of opium, acts with great energy on the animal economy. A grain and a half taken at three different times nearly proved fatal to a young man aged 17 years. 2. *Strychnia,* the active principle of the strychnos nux vomica, when introduced into the stomach, acts with extreme violence. It produces locked jaw in a very short time, and the animal is speedily destroyed. Half a grain of it in powder, blown into the throat of a rabbit, killed it in five minutes, and brought on the locked jaw in two. 3. *Brucia* is extracted from the bark of the brucea anti-dysenterica: its taste is exceedingly bitter, acrid, and durable in the mouth. Internally exhibited, it operates with a degree of intensity which is to that of strychnia as one to twelve. It excites tetanus, and acts upon the nerves without affecting the brain, or the intellectual faculties.

We shall now close our notice of this work by an extract from the introduction.

"The general reader will find, it is hoped, instruction blended with entertainment, in the articles *aërostation, air, climate, combustion, congelation, dew, electricity, equivalents, galvanism, geology, light, meteorolite, rain, and several other articles.*

"The agriculturist will find details not unworthy of his attention under the heads *absorbent, analysis of soils, carbonate, lime, manure, and soils.* Among the discussions interesting to manufacturers are, *acetic* and other *acids, alcohol, alum, ammonia, beer, bleaching, bread, caloric, coal, coal-gas, distillation, dying, ether, fat, fermentation, glass, ink, iron, ores, potash, pottery, salt, soap, soda, steel, sugar, and tanning.* Belonging to mineralogy, are the subjects *blowpipe, geology,* with its subordinate *rocks, ores, and meteorolite.*"

By the specimens which we have now placed under the observation of our readers, they will be prepared to estimate the merits of this scientific Dictionary. We may be allowed, in conclusion, to state the impression it has left on our own minds. The work, then, in our opinion is unrivalled; theory, in general, has been rejected from its pages; its doctrines and practical views are based on the results of experimental induction; and its style is, in a particular manner, significant, perspicuous, elegant. We, therefore, do cherish an agreeable anticipation of the excellence of Dr. Ure's forth-coming system of chemical philosophy.

